# CTLA4 +49AG (rs231775) and CT60 (rs3087243) gene variants are not associated with alopecia areata in a Mexican population from Monterrey Mexico^☆^^☆☆^

**DOI:** 10.1016/j.abd.2020.03.001

**Published:** 2020-03-20

**Authors:** Mauricio Andrés Salinas-Santander, Cristina Susana Cantu-Salinas, Jorge Ocampo-Candiani, Victor de Jesus Suarez-Valencia, Jennifer Guadalupe Ramirez-Guerrero, Celia Nohemi Sanchez-Dominguez

**Affiliations:** a Department of Investigation, Facultad de Medicina Unidad Saltillo, Universidad Autónoma de Coahuila, Saltillo, Coahuila, Mexico; b Dermatology Service, Hospital Universitario Dr José Eleuterio González, Universidad Autónoma de Nuevo León, Monterrey, Nuevo León, Mexico; c Department of Biochemistry and Molecular Medicine, Facultad de Medicina, Universidad Autónoma de Nuevo León, Nuevo León, Mexico

**Keywords:** Alopecia areata, Autoimmune diseases, Polymorphism, Single nucleotide

## Abstract

**Background:**

Alopecia areata is an autoimmune disease that produces non-scarring hair loss around the body. Gene variants of the cytotoxic T-lymphocyte antigen 4 (CTLA4) gene, a negative regulator of T-cell response, have been associated with a predisposition to autoimmune diseases in different populations; however, the involvement of these genetic variants in the development of AA is controversial.

**Objective:**

The present study evaluated the potential association of two CTLA4 gene variants with alopecia areata in a Mexican population.

**Methods:**

We genotyped +49AG (rs231775) and CT60 (rs3087243) variants in 50 AA patients and 100 healthy control participants through PCR-RFLP.

**Results:**

No statistical difference was observed for either of the gene variants regarding allele or genotype frequencies between AA patients and the controls when the parameters of family/personal history of autoimmune diseases or gender were considered (*p* > 0.05). *Study limitations:* Small sample size of patients and the data were obtained from Northeast Mexico population.

**Conclusion:**

The genetic variants rs231775 and rs3087243 of the CTLA4 gene are not a risk factor for the development of alopecia areata in the analyzed Mexican population.

## Introduction

Alopecia areata (AA) is an autoimmune disease that produces non-scarring hair loss through the body at any age.^1^ AA is commonly presented as single or multiple rounded patches, although complete baldness may occur, which is called AA totalis. If the hair loss occurs throughout the body is known as AA universalis. AA can also be presented as in the circumference of the head and is known as AA ophis.^2^ AA is considered a polygenic disease influenced by a genetically complex inheritance, especially of autoimmune and inflammatory related genes, along with environmental factors.^3^, ^4^ Various genes of the immune system have been reported to contribute to the development of this disease, such as the autoimmune regulator (AIRE) gene, human leukocyte antigen (HLA), interleukin (IL) genes, cytotoxic T lymphocyte-associated antigen 4 (CTLA4), protein tyrosine phosphatase non-receptor type 22 (PTPN22), tumor necrosis factor superfamily member 6 or CD95 (FAS) and FAS ligand (FASL) among others.^5^, ^6^

The cytotoxic T-lymphocyte antigen 4 (CTLA4) gene (2q34) is a member of the immunoglobulin superfamily encoding a protein whose function is that of a potent negative regulator of T-cell response. Also involved in the maintenance of immune tolerance, it has been implicated as a susceptibility gene in human autoimmune diseases, presenting several polymorphisms that have been associated with its development.^7^ Among the latter, two genetic variants may contribute to AA susceptibility: +49AG (rs231775), a missense variation leading to a threonine to alanine aminoacid substitution at codon 17 in the leader peptide (T17A); and CT60 (rs3087243), located 236 bp downstream of CTLA4 gene (3′-UTR).^8^ However, recent studies conducted in different populations have shown that the relationship of these genetic variants in the development of AA is controversial.^3^, ^9^

The current study sought to determine whether the CTLA4 +49AG (rs231775) and CT60 (rs3087243) gene variants constitute a predisposition factor for AA in a cohort of Mexican patients.

## Methods

### Selection of subjects and controls

The study included 50 unrelated Mexican subjects diagnosed with AA (32 females and 18 males; 30 ± 15 years old on average) and 100 healthy control participants (59 females and 41 males; 25 ± 8 years old on average), the latter showed a negative medical family history for autoimmune/inflammatory diseases. The patients were recruited from the Dermatology Department after the acceptance and signature of an informed consent. Ethical approval was given by the Institutional Review Board and this study was registered under the code DE09-001. Further details regarding the clinical and demographic characteristics, AA type and distribution of each patient were obtained during the clinical evaluation.

### DNA isolation and genotyping

Genomic DNA was isolated from peripheral venous blood using a “salting-out” method and suspended in Tris–EDTA (pH 7.8) at a final concentration 0.1–1.0 μg/μL before use. Genotype and allele frequencies of the CTLA4 +49AG (rs231775) andCT60 (rs3087243) gene variants were characterized by PCR-RFLP using a MJ Mini PTC1148 thermal cycler (Bio-Rad, Hercules; CA, USA), specific oligonucleotide primers (IDT, Coralville, IA, USA) and restriction enzymes (New England Biolabs, Ipswich, MA, USA) according to a previously published protocol.^10^ The fragments obtained were analyzed by electrophoresis in a 2.5% agarose gel containing ethidium bromide, visualized in UVP model 2UV High Performance Transilluminator (Upland, CA, USA) and documented.

### Statistical analysis

For genetic analysis, the sample size was calculated based on 2% worldwide alopecia areata incidence,^11^ assuming a power of 99.0% (a *Z* value of 2.33), a minimum number of 43 subjects is sufficient.

Data analysis was performed with the SPSS v21.0 software for windows (SPSS, Inc., Chicago, IL, USA) and the Epi-INFO TM 7 statistical program (Stone. Mountain, GA, USD Inc). Hardy–Weinberg equilibrium for the alleles was obtained using a goodness-of-fit test and the genotypic dependence between patients and controls was determined with a *χ*^2^ test; OR was calculated from 2 × 2 contingency tables (*p* < 0.05).

## Results

### Clinical characteristics of the participants

The present study analyzed the genotype distribution and allele frequency of CTLA4 +49AG and CT60 gene variants. Our universe (*n*) consisted of 50 AA patients and 100 control participants. The clinical presentation of AA in the patients was: 41 Patchy AA subjects (single patch *n* = 14, 8 females and 6 males; multiple patches *n* = 27, 19 females and 8 males), 4 Ophiasis AA subjects (3 females and 1 male), 1 female with Totalis AA and 4 Universalis AA subjects (3 males and 1 female). Ten patients (20%) had a personal history of autoimmune diseases (Vitiligo, type 2 DM, among others) and 36 subjects (72%) have family history of immune disease.

### Allele and genotype analysis

Several tests were performed for both genetic variants in order to assess the Hardy–Weinberg equilibrium (HWE) (Table 1) all of which had a *p* > 0.05, indicating that rs231775 and rs3087243 gene variants were in HWE in both AA patients and controls.

**Table 1:** Tests for deviation from Hardy–Weinberg equilibrium in AA patients and healthy controls.

SNP/HWE test	AA cases (*p*-value)	Controls (*p*-value)
*rs231775*		
Pearson	0.81	0.88
Likelihood-ratio	0.81	0.88
Fisher exact	0.78	1.00

*rs3087243*		
Pearson	0.89	0.50
Likelihood-ratio	0.89	0.50
Fisher exact	1.00	0.54

We compared the CTLA4 rs231775 and the rs3087243 alleles and genotypes of the control group with AA subjects (all AA types), and with the different types of AA, detailed results are shown in table 1. For CTLA4 rs231775, 30% of AA patients showed an homozygous wild type AA genotype, 48% showed the heterozygous AG genotype, and the remaining 22% were variant homozygous GG genotype. For CTLA4 rs3087243, 44% of the AA patients showed the homozygous wild type GG genotype, 44% showed the heterozygous AG genotype and the remaining 12% were variant homozygous AA genotype.

### Association of the genotypes in patients con alopecia areata

When CTLA4 rs231775 and rs3087243 genotypes and allele frequencies were compared between the whole cohort (all AA types) and controls, we observed that the heterozygous AG genotype for CTLA4 rs231775 and rs3087243 was more frequent in controls than across all AA patient groups. Further, no evidence was observed concerning the association of the allele variants and AA in the analyzed patients (*p* > 0.05) (Table 2). Furthermore, no relationship was found between genotype and type of AA with the personal/familiar history of autoimmune diseases or the sex of the analyzed patients (*p* > 0.05) (Table 3).

**Table 2:** Allele and genotype frequency of the gene variants CTLA4 +49AG (rs231775) and CT60 (rs3087243) in AA patients and healthy controls.

Genotype	AA patients no. (%)	Controls no. (%)	*χ*^2^	OR	95% CI	*p*-Value
*All AA types*	rs231775					
AA	15 (30)	28 (28.3)	0.086			0.958
AG	24 (48)	50 (50.5)				
GG	11 (22)	21 (21.2)				
AG + GG	24 (48) + 11 (22)	50 (50.5) + 21 (21.2)	0.048	1.087	0.515–2.292	0.827

*Alleles*						
A	54 (54)	106 (53.5)	0.006	1.019	0.629–1.650	0.939
G	46 (46)	92 (46.5)	0.006	0.982	0.606–1.590	0.939

*Patchy AA*						
AA	13 (31.7)	28 (28.3)	0.225			0.893
AG	19 (46.3)	50 (50.5)				
GG	9 (22)	21 (21.2)				
AG + GG	19 (46.3) + 9 (22)	50 (50.5) + 21 (21.2)	0.164	1.177	0.534–2.594	0.685

*Alleles*						
A	45 (54.9)	106 (53.5)	0.042	1.056	0.630–1.770	0.837
G	37 (45.1)	92 (46.5)	0.042	0.947	0.565–1.589	0.837

*Multiple patch AA*						
AA	11 (40.7)	28 (28.3)	1.66			0.436
AG	12 (44.5)	50 (50.5)				
GG	4 (14.8)	21 (21.2)				
AG + GG	12 (44.5) + 4 (14.8)	50 (50.5) + 21 (21.2)	1.541	1.743	0.721–4.218	0.215

*Alleles*						
A	34 (63)	106 (53.5)	1.527	1.476	0.795–2.740	0.217
G	20 (37)	92 (46.5)	1.527	0.678	0.365–1.259	0.217

*All AA types*	rs3087243					
AA	6 (12)	16 (16)	2.129			0.345
AG	22 (44)	52 (52)				
GG	22 (44)	32 (32)				
AG + GG	22 (44) + 22 (44)	52 (52) + 32 (32)	0.426	0.716	0.262–1.959	0.514

*Alleles*						
A	34 (34)	84 (42)	1.788	0.711	0.432–1.173	0.181
G	66 (66)	116 (58)	1.788	1.406	0.853–2.318	0.181

*Patchy AA*						
AA	6 (14.6)	16 (16)	1.168			0.558
AG	18 (43.9)	52 (52)				
GG	17 (41.5)	32 (32)				
AG + GG	18 (43.9) + 17 (41.5)	52 (52) + 32 (32)	0.041	0.9	0.325–2.49	0.839

*Alleles*						
A	30 (36.6)	84 (42)	0.708	0.797	0.469–1.353	0.4
G	52 (63.4)	116 (58)	0.708	1.255	0.739–2.132	0.4

*Multiple patch AA*						
AA	4 (14.8)	16 (16)	0.031			0.985
AG	14 (51.9)	52 (52)				
GG	9 (33.3)	32 (32)				
AG + GG	14 (51.9) + 9 (33.3)	52 (52) + 32 (32)	0.023	0.913	0.278–2.998	0.881

*Alleles*						
A	22 (40.7)	84 (42)	0.028	0.949	0.515–1.749	0.868
G	32 (59.3)	116 (58)	0.028	1.053	0.572–1.941	0.868

**Table 3:** Analysis of the gene variants CTLA4 +49AG (rs231775) and CT60 (rs3087243) frequencies with the sex and personal/familiar history of autoimmune diseases.

	AA	AG	GG	*p*-Value	AA	AG	GG	*p*-Value
*Sex*								
Female (%)	8 (25%)	16 (50%)	8 (25%)	0.555	4 (12.5%)	14 (43.75%)	14 (43.75%)	0.990
Male (%)	7 (38.9%)	8 (44.4%)	3 (16.7%)		2 (11.2%)	8 (44.4%)	8 (44.4%)	

*Personal history of autoimmune diseases*								
Yes (%)	3 (30%)	3 (30%)	4 (40%)	0.261	1 (10%)	3 (30%)	6 (60%)	0.515
No (%)	12 (30%)	21 (52.5%)	7 (17.5%)		5 (12.5%)	19 (47.5%)	16 (40%)	

*Familiar history of autoimmune diseases*								
Yes (%)	10 (27.8%)	19 (52.8%)	7 (19.4%)	0.548	4 (11.1%)	17 (47.2%)	15 (41.7%)	0.761
No (%)	5 (38.4%)	4 (30.8%)	4 (30.8%)		2 (14.3%)	5 (35.7%)	7 (50%)	

## Discussion

AA is an autoimmune hair-loss condition that has been associated with multiple gene variants, several of which participate in immune response pathways related to HLA.^12^ The CTLA4 gene product is a receptor expressed by CD4+ and CD8+ T-cells. Experiments in knockout mice have demonstrated the important role that CTLA4 plays in protection against autoimmunity. CTLA-4 is a critical negative regulator of T cell responses, and the action of CTLA4 on Treg cells can control the activity of other cells and controlling fatal autoimmunity.^13^

The association between the presence of the CTLA4 gene variants and the development of different autoimmune diseases has been suggested.^14^, ^15^ However in immune dermatological diseases, the results have been controversial; in vitiligo their participation or lack of influence^16^ has been seen. A similar situation has been observed with the development of psoriasis.^17^, ^18^

In this regard, considering the possible contribution of the autoimmune function in alopecia, genetic variant studies of CTLA4 gene have been explored in diverse populations. A previous association analysis has suggested the plausible involvement of CTLA4 gene variants as a risk factor in the development of AA in a European population,^8^ with a stronger effect in patients with severe forms of the disorder.

Another study conducted on an Italian population, where rs231775 and rs3087243 gene variants were analyzed, observed the latter as a risk factor in the development of patchy AA.^9^ On the other hand, a study conducted on Iran nationals failed to prove the association of the rs3087243 gene variant in the development of AA.^3^

Previously, we observed the participation of genetic variants of TNFα and PTPN22^19^ as a risk factor for AA in Mexican patients; both of these genes’ function is related to the regulation of immune mechanisms. However, the participation of CTLA4 genetic variants in AA has not been analyzed in this population.

Some SNPs within the CTLA-4 gene have already been analyzed for susceptibility to rheumatoid arthritis (RA) in the Mexican population; among them, rs5742909, rs231775 and rs3087243, suggesting that the −319C/+49G/CT60G haplotype of the CTLA-4 gene is a risk factor for RA, whereas that the rs3087243 SNP could be a protective factor against this type of autoimmune disease.^20^

However, no significant differences were observed for rs231775 and rs3087243 in our study population (Table 2), suggesting that these variants are not a risk factor in the development of AA or Patchy AA in the Mexican population.

## Conclusion

In conclusion, the genetic variants rs231775 and rs3087243 of the CTLA4 gene do not constitute a risk factor in the development of AA in the Mexican population of Monterrey, Mexico. Further, these genetic variants have no association between family/personal backgrounds of autoimmune diseases or the gender of the study subjects.

## Financial support

None declared.

## Authors' contributions

Mauricio Andrés Salinas Santander: Statistic analysis; approval of the final version of the manuscript; conception and planning of the study; elaboration and writing of the manuscript; obtaining, analysis, and interpretation of the data; effective participation in research orientation; critical review of the literature; critical review of the manuscript.

Cristina Susana Cantu-Salinas: Approval of the final version of the manuscript; conception and planning of the study; elaboration and writing of the manuscript; obtaining, analysis, and interpretation of the data; effective participation in research orientation; critical review of the literature; critical review of the manuscript.

Jorge Ocampo-Candiani: Approval of the final version of the manuscript; conception and planning of the study; effective participation in research orientation; critical review of the literature; critical review of the manuscript.

Victor de Jesus Suarez-Valencia: Approval of the final version of the manuscript; elaboration and writing of the manuscript; obtaining, analysis, and interpretation of the data; critical review of the literature; critical review of the manuscript.

Jennifer Guadalupe Ramirez-Guerrero: Approval of the final version of the manuscript; elaboration and writing of the manuscript; obtaining, analysis, and interpretation of the data; critical review of the literature.

Celia Nohemi Sanchez-Dominguez: Approval of the final version of the manuscript; elaboration and writing of the manuscript; critical review of the literature; critical review of the manuscript.

## Conflicts of interest

None declared.
